# Tongue Tumor Detection in Medical Hyperspectral Images

**DOI:** 10.3390/s120100162

**Published:** 2011-12-23

**Authors:** Zhi Liu, Hongjun Wang, Qingli Li

**Affiliations:** 1 School of Information Science and Engineering, Shandong University, Jinan 250100, China; E-Mail: hongjunwang1962@gmail.com; 2 School of Information Science and Technology, East China Normal University, Shanghai 200062, China; E-Mail: qinglili7609@gmail.com

**Keywords:** tongue tumor detection, sparse representation, medical hyperspectral imaging

## Abstract

A hyperspectral imaging system to measure and analyze the reflectance spectra of the human tongue with high spatial resolution is proposed for tongue tumor detection. To achieve fast and accurate performance for detecting tongue tumors, reflectance data were collected using spectral acousto-optic tunable filters and a spectral adapter, and sparse representation was used for the data analysis algorithm. Based on the tumor image database, a recognition rate of 96.5% was achieved. The experimental results show that hyperspectral imaging for tongue tumor diagnosis, together with the spectroscopic classification method provide a new approach for the noninvasive computer-aided diagnosis of tongue tumors.

## 1. Introduction

Cancer of the tongue is a malignant tumor that begins as a small lump, a firm white patch, or an ulcer. If untreated, the tumor may spread throughout the tongue and to the gums. As the tumor grows, it becomes increasingly life threatening by metastasizing to lymph nodes in the neck and to the rest of the body. Early detection has an immense effect on outcome because cancer treatment is often simpler and more effective when diagnosed at an early stage. Tumor detection methods may help physicians diagnose cancers, to dissect the malignant region with a safe margin, and to evaluate the tumor bed after resection. Currently, histopathology is still the gold standard for cancer diagnosis. However, this method is invasive, expensive, greatly depends on the judgment of pathologists, and needs time for preparing the results. Moreover, the biopsy specimens can only be captured from a few points. Therefore, a simple, noninvasive, and reliable technique for rapid cancer detection is required to aid physicians.

Computer vision technologies provide an approach to computer-aided diagnosis assisted by digital cameras. Conventional color cameras acquire color intensity from three broad spectral visible bands, *i.e.*, red, green, and blue. However, the actual information from these three bands is very limited [1].

Hyperspectral image (HSI) sensors generate two-dimensional spatial images along a third spectral dimension. Each pixel in the hyperspectral image has a sequence of reflectance in different spectral wavelengths that display the spectral signature of that pixel; this indicates that this kind of sensor measures the intensity over a hundred or more narrow spectral bands. Currently, hyperspectral imaging has been used for medicine, where it is known as medical hyperspectral imaging (MHSI). MHSI is a novel, camera-based method of imaging spectroscopy that integrates spatial and spectroscopic data from tissue in a set of images. MHSI delivers near-real-time images of biomarkers in tissue, thereby providing an assessment of pathophysiology and the potential to distinguish different tissues based on their spectral characteristics. Therefore, MHSI is a promising method for noninvasive, rapid, and inexpensive evaluation of cancer in the tumor bed at the time of diagnosis [2].

There have been several previous studies on MHSI in the last decade [3]. Panasyuk used MHSI in distinguishing tumors from normal breast and other tissues, which demonstrated a sensitivity of 89% and a specificity of 94% for the detection of residual tumors [4]. Akbari [5] detected gastric cancer by MHSI. Klaessens *et al*. [6] measured the changes in O_2_Hb and HHb concentration in tissues. Liu [7] and Li [8] used MHSI for tongue diagnosis in Traditional Chinese Medicine. Marzani [9] used an artificial neural network-based multispectral imaging system to reconstruct the hyperspectral cutaneous data. Novakovic [10] presented their work on the phototherapy of psoriasis based on spectrophotometric intracutaneous analysis. Medina [11] worked on the human iris *in vivo* by MHSI. Larsen [12] studied atherosclerotic plaques by MHSI. All these research results demonstrate that MHSI has tremendous potential for detecting important biomarkers based on their unique spectral signatures during the early stages of disease. However, some bottlenecks have limited its use for *in vivo* screening applications, most notably their huge temporal cost and poor spatial resolution. In addition, their sensitivity and accuracy need to be improved. For tongue cancer, because of the instinctive squirming of the human tongue and the noise caused by saliva, detecting the tumor range accurately is difficult under MHSI. To address this problem, we present an MHSI system based on an acousto-optic tunable filter (AOTF) and the corresponding classification algorithm based on sparse representation (SR). The rest of this paper is organized in four sections. Section 2 presents the data acquisition of the proposed system. Section 3 introduces the proposed method for cancer area detection. Section 4 then presents the experiments conducted to evaluate the performance of the proposed system. Finally, concluding remarks are offered in Section 5.

## 2. Hyperspectral Image Acquisition

Over the last years, hyperspectral imaging devices have been mainly based on two sequential acquisition principles. The first is wavelength scanning; single images are recorded for each different wavelength by many discrete filters or tuneable filters. The second is spatial scanning, which requires relative movement between camera and sample [13]. For medical applications, especially for tongue tumor detection, tuneable filters are preferable because they are fast and versatile, and do not require any mechanically moveable parts. Furthermore, the spatial resolution of HSI systems is (within limits) independent of the tuneable filter and can be optimized by selecting optics and cameras [14]. AOTF is a rapid wavelength-scanning solid-state device that operates as a tunable optical band pass filter. The acoustic wave is generated by radiofrequency signals, which are applied to the crystal via an attached piezoelectric transducer [15].

**Figure 1.**
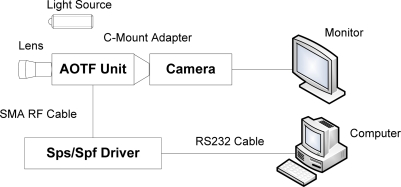
The schematic diagram of the system.

AOTF is based on the acoustic diffraction of light in an anisotropic medium and it has several advantages over traditional spectrometers, which are typically based on a filter wheel or a grating, and therefore require careful handling and frequent calibration. They also suffer from lower scan speeds and lower reliability. AOTFs are solid-state tunable filters with no moving parts and are therefore immune to orientation changes or even severe mechanical shock and vibrations. Moreover, AOTFs are high-throughput and high-speed programmable devices capable of accessing wavelengths at rates of up to 100 kHz, making them excellent tools for spectroscopy. Other advantages of AOTF technology are their broad tuning range (0.4–5 μm), large field of view, and the fact that they are electronically programmable. Image capture can achieve real-time acquisition (30 frames per second or much faster). Unlike grating-based instruments, no motion of the imager or object is required to obtain a complete image cube. This feature makes the structure of the new AOTF-based system simpler and more compact. A schematic diagram of the proposed system for tongue tumor detection is shown in Figure 1.

**Figure 2.**
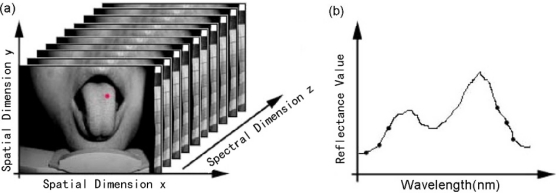
(**a**) The hyperspectral image cube; (**b**) the spectrum corresponding to the red point in (a) [16].

The hyperspectral imaging system consists of a spectral illuminator, which provides spectroscopic light to the sample and a focal plane array detector, which captures the reflected spatial image information, synchronized by a computer program. The AOTF unit is a VA210, with a wavelength range of 600–1,000 nm (Brimrose Corporation, USA), controlled by a PC controlled RF Driver, which is handled by the computer through the RS232 cable. The camera is a JAI BM141-GE with a GigE port and a 1,392 × 1,040 array with 6.45 μm × 6.45 μm pixels, a frame rate of 30 frames/s at full resolution in continuous operation, which provides good performance for transferring the huge amount image data. 81 mono-channel images with 5 nm spectral resolution were used for tongue tumor detection. A pair of 500 W halogen lamps, which can provide a fairly uniform lighting of the subject, were used as light sources for illumination. Computer software was specifically developed by the authors for managing the spectral illumination turn on/off, data collection, image processing, and classification. The optical part of the system and a standard Tamron 28–80 mm f//3.5 lens are mounted in front of the AOTF unit. The light source, battery backup systems, and power supplies are placed on a cart, which provides the system with portability within and between surgical and clinical suites. Using this system, every point on the surface of the tongue is represented on the matrix detector by a series of monochromatic points that produces a continuous spectrum in the direction of the spectral axis, which is illustrated in Figure 2.

## 3. Method for Tumor Detection

**Figure 3.**
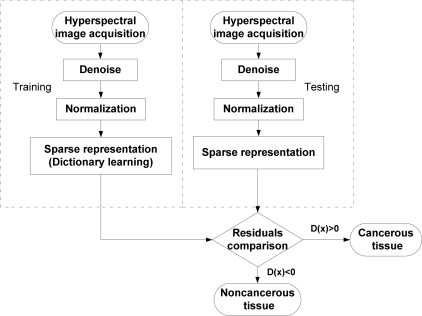
Flowchart of the method.

In this section, the method for tumor detection in medical hyperspectral images based on SR is presented. First, the preprocessing on the hyperspectral image is introduced. Second, the details of the sparse representation model used in the proposed algorithm are described. Finally, SR is extended for tumor target detection. The overview of the method is shown in Figure 3.

The method includes two stages: training and testing. For training, the hyperspectral images of the tongue tumor are collected to learn the dictionary for SR after the denoising module and normalization module. Similar to the training stage, the test sample also has a sparse representation after denoising and normalization. Then, the reconstruction residuals are computed for comparison to get the decision. The following subsection describes these steps in detail.

### 3.1. Preprocessing

The preprocessing step mainly includes the denoising and normalization. For tongue tumor detection using a hyperspectral imager, the noise is introduced because of the saliva on the surface of the tongue and its instinctive squirming. A median filter is used to remove the noise effects.

Normalization of the hyperspectral image data is necessary to eliminate the influence of the dark current. A standard reference white board was placed in the scene of imaging, and its data were utilized as the white reference. This white reference is a standard reflectance that should be used for data normalization, which shows the maximum standard reflectance in each wavelength and in the capturing of time temperature. The reflectance from the board provides an estimate of the incident light on the tongue at each wavelength, which is used in the normalization of the spectrum. The dark current was captured by keeping the camera shutter closed. Then the data were normalized to determine the relative reflectance using the following equation:

(1) $$R(\lambda )=\frac{I_{\mathrm{raw}}\;(\lambda )-I_{\mathrm{dark}}\;(\lambda )}{I_{\mathrm{white}}\;(\lambda )-I_{\mathrm{dark}}\;(\lambda )}$$

where *R*(*λ*) is the calculated relative reflectance value for each wavelength, *I_raw_* (*λ*) is the raw data radiance value of a given pixel, and *I_dark_* (*λ*) and *I_white_* (*λ*) are the dark current and the white board radiance acquired for the spectral band of the sensor, respectively.

### 3.2. Sparse Representation

SR has proven to be an extremely powerful tool for representing and compressing high-dimensional signals. This success is mainly because important classes of signals, such as audio and image signals, have natural sparse properties [17]. SR is able to extract the simple but important properties of the data. SR has been used for face recognition in gray images successfully [18]. Recently, Chen [19] has extended SR for classification in hyperspectral images. For completeness, SR is briefly introduced as follows.

Suppose that there are *L* image classes and *n* training images with *p* × *q* pixels. Each image can be represented as a column vector with D = *p* × *q* dimensionality. This means that the image with *p* × *q* pixels can be represented as a column vector with *p* × *q* dimensionality by concatenating each column of the image. Let ***A****_k_* = [*x*_*k*1_,…,*x_kn_k__*] be a *D* × *n_k_* matrix of training images from the *k^th^* class with *n_k_* training samples. ***A****_k_* is called a subdictionary matrix. Matrix ***A*** is defined as the concatenation of the subdictionary from all the classes as:

(2) $$\begin{array}{ll} A & =\left[A_{1},\ldots ,A_{L}\right]\;\in \;R^{D\times (\mathrm{nL})}\\ & \;\;\;=\left[x_{11},\ldots ,x_{1n_{1}}|x_{21},\ldots ,x_{2n_{2}}|\ldots |x_{L1},\ldots ,x_{Ln_{k}}\right] \end{array}$$

A test vector *y* = *R_D_* from an unknown class, which can be represented by a linear combination of the training vectors as:

(3) $$y=\sum_{i=1}^{L}\;\sum_{j=1}^{n}\;a_{\mathrm{ij}}\;x_{\mathrm{ij}}$$

where *a_ij_* ∈ *R* are the coefficients. Equation (3) can be written as:

(4) $$y=A\bar{\alpha }$$

where ***ᾱ*** = [*α*_11_,…,*α*_1*n*_1__|*α*_21_,…,*α*_2*n*_2__|…|*α*_*L*1_,…,*α_Ln_k__*] is the coefficient vector. Thus, any test image *y* that belongs to the same class can be approximately represented by the linear span of the training samples from the corresponding class *k*. This means that most of the coefficients not associated with class *k* in ***ᾱ*** will be close to zero. The training samples from the same class as the test sample have non-zero coefficients in the linear combination, whereas those from a different class from the test sample have zero coefficients. Hence, ***ᾱ*** is a sparse vector. If a test sample is from the *i^th^* class, the coefficient vector ***ᾱ*** of the training samples should be:

(5) $$\bar{\alpha }=\left[0,\ldots ,0,\;\alpha_{i1},\ldots ,\alpha_{{\mathrm{in}}_{i}},\;0,\ldots ,0\right]$$

The behavior of a linear system is determined by the relationship between the number of columns of ***A*** (the number of unknowns) and the number of rows of ***A*** (the number of equations). When the system has fewer equations than unknowns, for example, *D* < *nL* in dictionary ***A*** ∈ *R*^*D*×(*nL*)^, it may have an infinite number of solutions [17]. As a result, in all solutions of *y* = ***Ax***, arriving at the best solution is possible, which is infinitely close to the ideal solution. The sparsest solution of *y* = ***Ax*** is defined as the following optimization problem:

(6) $$\hat{\alpha }=\text{arg}\;\underset{\bar{\alpha }}{\text{min}}{\left\|\bar{\alpha }\right\|}_{1}\;\;\text{subject to}\;y=A\bar{\alpha }$$

where ‖·‖_1_ denotes the *l*_1_ norm. This problem is often known as basis pursuit and can be solved in polynomial time [20]. The *l*_1_ norm is an approximation of the *l*_0_ norm. The approximation is necessary because the optimization problem in Equation (6) with the *l*_0_ norm, which is used to seek the sparsest ***ᾱ***, is NP-hard and computationally difficult to solve [21]. Considering that noise is inevitable in natural images, Equation (6) can be written as:

(7) $$\hat{\alpha }=\text{arg}\;\underset{\bar{\alpha }}{\text{min}}\;{\left\|\bar{\alpha }\right\|}_{1}\;\;\text{subject to}\;{\left\|y-A\bar{\alpha }\right\|}_{2}\;\leq \varepsilon$$

where *ε* is the error tolerance. Therefore, the test sample can be represented as:

(8) $$y=A\bar{\alpha }+\eta$$

where ‖*η*‖_2_ ≤ *ε*.

### 3.3. Tumor Detection Based on SR

Based on the work by Chen [19], a method for tumor detection in MHSI is proposed. Let *x* be a hyperspectral pixel observation, which is a B-dimensional vector whose entries correspond to the spectral bands with *B* being the number of spectral bands. In the hyperspectral images of the tongue, if *x* is a noncancerous pixel, its spectrum approximately lies in a low-dimensional subspace spanned by the noncancerous training samples. Then, *x* can be approximately represented by a linear combination of the training samples as follows:

(9) $$\begin{array}{ll} x & =\alpha_{1}a_{1}^{\mathrm{nc}}+\alpha_{2}a_{2}^{\mathrm{nc}}+\cdots +\alpha_{N_{\mathrm{nc}}}a_{N_{\mathrm{nc}}}^{\mathrm{nc}}=\underset{A_{\mathrm{nc}}}{\underset{︸}{\left[a_{1}^{\mathrm{nc}}\;a_{2}^{\mathrm{nc}}\;\;\cdots a_{N_{\mathrm{nc}}}^{\mathrm{nc}}\right]}}\underset{\bar{\alpha }}{\underset{︸}{{\left[\alpha_{1}\;\alpha_{2}\cdots \alpha_{N_{\mathrm{nc}}}\right]}^{T}}}\\ & =A_{\mathrm{nc}}\bar{\alpha } \end{array}$$

where *N_nc_* is the number of noncancerous training samples, ***A****_nc_* is the *B* × *N_c_* noncancerous dictionary consisting of the noncancerous training pixels, and ***ᾱ*** is a sparse vector whose entries contain the abundances of the corresponding atoms in ***A****_nc_*.

A cancerous pixel *x* can be sparsely represented by a linear combination of the training samples:

(10) $$\begin{array}{ll} x & =\beta_{1}a_{1}^{c}+\beta_{2}a_{2}^{c}+\cdots +\beta_{N_{c}}\;a_{N_{c}}^{c}=\underset{A_{c}}{\underset{︸}{\left[a_{1}^{c}\;\;\;a_{2}^{c}\;\;\;\cdots a_{N_{c}}^{c}\right]}}\underset{\bar{\beta }}{\underset{︸}{{\left[\beta_{1}\;\;\;\beta_{2}\cdots \beta_{N_{c}}\right]}^{T}}}\\ & =A_{c}\bar{\beta } \end{array}$$

where *N_c_* is the number of cancerous training samples, ***A***_*c*_ is the *B* × *N_c_* cancerous dictionary consisting of the cancerous training pixels, and ***β̄*** is a sparse vector whose entries contain the abundances of the corresponding atoms in ***A****_c_*.

A test sample lies in the union of the noncancerous and cancerous subspaces. Therefore, by combining the two dictionaries ***A****_nc_* and ***A****_c_*, a test sample *x* can be written as a sparse linear combination of all training pixels:

(11) $$x=A_{\mathrm{nc}}\;\bar{\alpha }+A_{c}\;\bar{\beta }=[\underset{A}{\underset{︸}{A_{\mathrm{nc}}\;\;A_{c}}}]\underset{\bar{\gamma }}{\underset{︸}{\left[\begin{array}{c} \alpha \\ \beta \end{array}\right]}}=A\bar{\gamma }$$

where ***A*** is a *B*×(*N_nc_* + *N_c_*) matrix consisting of both noncancerous and cancerous training samples, and ***γ̄*** is a (*N_nc_* + *N_c_*) -dimensional vector formed by concatenating the two sparse vectors ***ᾱ*** and ***β̄***, which is also a sparse vector.

As discussed above, a test sample can be approximately represented by very few training samples. Given the dictionary of training samples ***A*** = [***A****_nc_* ***A****_c_*], the representation ***γ̄*** that satisfies *x* = ***Aγ̄*** can be obtained by solving the following optimization problem for the sparsest vector:

(12) $$\hat{\gamma }=\text{argmin}{\left\|\gamma \right\|}_{0}\;\text{subject to}\;x=A\bar{\gamma }$$

If the solution is sparse enough, the optimization problem in Equation (12) can be solved efficiently as a linear programming [20] or by greedy pursuit algorithms [22,23].

Once the sparse vector is obtained, the class of *x* can be determined by comparing the residuals *γ_nc_*(*x*) = ‖*x* − ***Ā****_nc_****α̂***‖_2_ and *γ_c_*(*x*) = ‖*x* − ***Ā****_c_****β̂***‖_2_, where ***α̂*** and ***β̂*** represent the recovered sparse coefficients that correspond to the noncancerous and cancerous dictionaries, respectively. In the proposed approach, the algorithm output is calculated by

(13) $$D(x)=\text{lg}\;\frac{r_{\mathrm{nc}}\;(x)}{r_{c}\;(x)}=\text{lg}\frac{{\left\|x-{\bar{A}}_{\mathrm{nc}}\hat{\alpha }\right\|}_{2}}{{\left\|x-{\bar{A}}_{c}\hat{\alpha }\right\|}_{2}}$$

If *D*(*x*) > 0, then *x* is determined as a cancerous pixel; otherwise, *x* is labeled as noncancerous.

## 4. Experimental Results and Analysis

**Figure 4.**
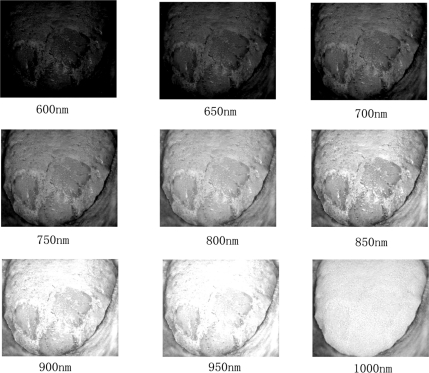
Some examples of tongue tumor hyperspectral images.

To the best of our knowledge, there is no public medical hyperspectral image database. Therefore, we constructed our own tongue tumor image database. The current database includes 65 tumors and performed partial resection of 34 tumors, which yields 34 full tumor/partial resection/tumor bed sets for analysis. For performance evaluation, both the results under MHSI and histopathology were recorded. Figure 4 presents examples of tongue tumor hyperspectral images in the database.

**Figure 5.**
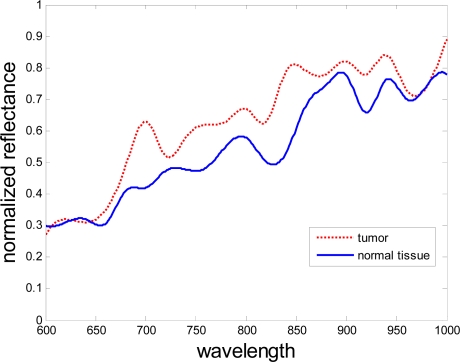
Reflectance spectra. Tumor pixels are shown in red and normal pixels are shown in blue.

Each pixel in the hyperspectral image has a sequence of brightness in different wavelengths, which constructs the spectral signature of that pixel. The difference in spectral signature between the tumor and the normal tissue can be determined. The curves in Figure 5 show the difference in spectral signatures between normal and cancerous tissues. The values are the averages of the reflectance of the pixels from the normal and cancerous tissue regions. The curves are smoothed for a clear image. The standard deviation in each wavelength is shown in Figure 6. In this figure, the red squares and blue circles represents the standard deviation of the reflectence values of the pixels from the normal tissue region and the cancerous tissue region respectively. The differences between the spectral signatures are strongly related to the protein changes, as shown in the paper by Tsenkova [24].

SR classifier is used to detect the cancerous tissues based on the spectral signature. As the spectral signatures of normal tissues are different from cancerous ones, 81 bands were used without compression to separate the normal from the cancerous parts. After this step, majority of the pixels were detected, although there were some that were lost because of glare. To address this problem, we used mathematical morphology method as a post processing step to fill the holes.

**Figure 7.**
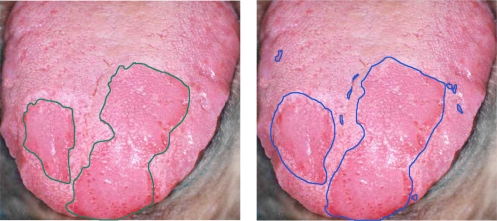
Tumor region. Expert labeling (**left**) and classifier prediction of tumor regions (**right**).

In this study, to lend credibility to the performance analysis of the system, histopathologic analysis was performed by a physician on each sample and confirmed normal and malignant tissue locations as the basis for comparison. Figure 7 shows the performance of the detection using the proposed method based on SR. As shown in the figure, the system was able to identify the same cancerous regions as the medical expert. To evaluate the performance of our method, we randomly chose around 10% of the labeled samples for training and 90% for testing. The number of training and test samples for each class is shown in Table 1.

**Figure 8.**
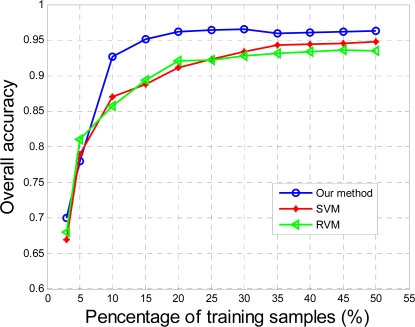
Effect of the number of training samples.

The proposed method, as well as the classical methods, e.g., support vector machine (SVM) [25], relevance vector machine (RVM) [26], were applied to the MHSI for tongue tumors and the results were compared quantitatively by the curves shown in Figure 8. The graph describes the probability of detection as a function of the percentage of training samples. In Figure 8, the proposed method based on a 2D medium filter and SR outperform the other two popular methods with 96.5% accuracy. The method worked well even in tumors up to a depth < 3 mm and was covered with mucosa [5].

This SR-based method, unlike SVM and RVM, search for dedicated atoms in the training dictionary for each test pixel (*i.e.*, the support of the sparse vector is dynamic). Therefore, the sparsity-based algorithms are more computationally intensive than SVM and RVM. The computer used in this system was equipped with an Intel^®^ CPU i7 with a 4 GB random access memory. The comparison in terms of speed of classification is shown in Table 2. As shown, SR achieves much faster classification times than the other popular methods.

The performance criteria for cancer detection were the false negative rate (FNR) and the false positive rate (FPR), which were calculated for each hyperspectral image. When a pixel was not detected as a tumor pixel, the detection was considered as false negative if the pixel was an actual tumor pixel in the pathological results. FNR refers to the number of false negative pixels divided by the total number of tumor pixels. When a pixel was detected as a tumor tissue, the detection was a false positive if the pixel was not a tumor. FPR refers to the number of false positive pixels divided by the total number of normal tissues. The numerical results of the FPR and FNR and a comparison among our method, SVM, and RVM is given in Table 3.

## 5. Conclusions

A hyperspectral image system for tongue tumor detection based on AOTF technology has been presented. Algorithms based on the spectral characteristics of the tissues and sparse representation, were proposed to distinguish between tumors and normal tissues. The capability of the system has been proven through an MHSI dataset. A best recognition rate of 96.5% was achieved. The experimental results demonstrate that the system has great potential as an important imaging technology for medical imaging devices that provide additional diagnostic information regarding tissues under investigation. Although the final diagnostic decision remains the burden of physicians, the system supports physicians during decision making.

Follow-up studies on patients are planned to allow the quantitative grading of tumors automatically according to their clinicopathologic features and to study further the spectrochemical properties of tumor tissues. This system has obvious applications as a computer-aided medical diagnostic tool. The modality of imaging combined with spectroscopic data will prove useful in tumor detection and in the assessment of tissue response to therapy.

## Figures and Tables

**Figue 6.**
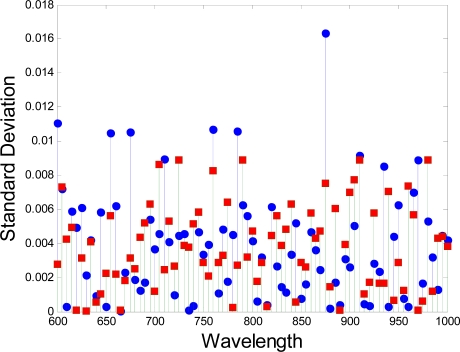
The standard deviation in each wavelength.

**Table 1.** The two classes (noncancerous and cancerous) and the training and test set for each class.

**Class**		**Samples**	

**No.**	**Name**	**Train**	**Test**
1	noncancerous	954	8,609
2	cancerous	796	7,237

**Table 2.** Classification time for different methods on tumor detection.

**Methods**	**Our method**	**SVM**	**RVM**
Classification time (s)	3.4	7.8	6.9

**Table 3.** Evaluation results with FPR and FNR.

**Methods**	**Our method (%)**	**SVM (%)**	**RVM (%)**
FPR	6.3	12.5	10.9
FNR	8.7	15.2	13.5
